# Policies for biosimilar uptake in Europe: An overview

**DOI:** 10.1371/journal.pone.0190147

**Published:** 2017-12-28

**Authors:** Evelien Moorkens, Arnold G. Vulto, Isabelle Huys, Pieter Dylst, Brian Godman, Simon Keuerleber, Barbara Claus, Maria Dimitrova, Guenka Petrova, Ljiljana Sović-Brkičić, Juraj Slabý, Robin Šebesta, Ott Laius, Allan Karr, Morgane Beck, Jaana E. Martikainen, Gisbert W. Selke, Susan Spillane, Laura McCullagh, Gianluca Trifirò, Patricia Vella Bonanno, Asbjørn Mack, Antra Fogele, Anita Viksna, Magdalena Władysiuk, Helder Mota-Filipe, Dmitry Meshkov, Marija Kalaba, Simona Mencej Bedrač, Jurij Fürst, Corrine Zara, Peter Skiöld, Einar Magnússon, Steven Simoens

**Affiliations:** 1 KU Leuven Department of Pharmaceutical and Pharmacological Sciences, Leuven, Belgium; 2 Hospital Pharmacy, Erasmus University Medical Center, Rotterdam, The Netherlands; 3 Medicines for Europe, Brussels, Belgium; 4 Department of Laboratory Medicine, Karolinska Institutet, Stockholm, Sweden; 5 Strathclyde Institute for Pharmacy and Biomedical Sciences, University of Strathclyde, Glasgow, United Kingdom; 6 Main Association of Austrian Social Security Institutions, Vienna, Austria; 7 Faculty of pharmaceutical sciences, Ghent University, Ghent, Belgium; 8 Faculty of Pharmacy, Medical University of Sofia, Sofia, Bulgaria; 9 Croatian Health Insurance Fund, Zagreb, Croatia; 10 State Institute for Drug Control, Prague, Czech Republic; 11 State Institute for Drug Control, Brno, Czech Republic; 12 State Agency of Medicines, Tartu, Estonia; 13 Department of Traumatology and Orthopedics, University of Tartu, Tartu, Estonia; 14 Karr Consultancy Ltd, Hertfordshire, United Kingdom; 15 OMEDIT Alsace, Agence Régionale de Santé du Grand Est, Strasbourg, France; 16 Research Unit, Social Insurance Institution, Helsinki, Finland; 17 Wissenschaftliches Institut der AOK (WIdO), Berlin, Germany; 18 Department of Pharmacology and Therapeutics, Trinity College Dublin, Dublin, Ireland; 19 National Centre for Pharmacoeconomics, St James's Hospital, Dublin, Ireland; 20 Department of Biomedical and Dental Sciences and Morphofunctional Imaging, University of Messina, Messina, Italy; 21 Strathclyde Institute for Pharmacy and Biomedical Sciences, University of Strathclyde, Glasgow, United Kingdom; 22 Division Pharmaceuticals, Norwegian Hospital Procurement Trust, Oslo, Norway; 23 Department of Medicines and Medical Devices, The National Health Service, Riga, Latvia; 24 HTA Consulting, Cracow, Poland; 25 Faculty of Pharmacy, University of Lisbon, Lisbon, Portugal; 26 Semashko National Research Institute for Public Health, Moscow, Russia; 27 Primary healthcare center Zemun, Belgrade, Serbia; 28 Agency for Medicinal Products and Medical Devices of the Republic of Slovenia, Ljubljana, Slovenia; 29 Health Insurance Institute, Ljubljana, Slovenia; 30 Barcelona Health Region, Catalan Health Service, Barcelona, Spain; 31 TLV, Stockholm, Sweden; 32 Ministry of Welfare, Reykjavik, Iceland; Jagiellonian University, POLAND

## Abstract

**Background:**

Across European countries, differences exist in biosimilar policies, leading to variations in uptake of biosimilars and divergences in savings all over Europe.

**Objectives:**

The aim of this article is to provide an overview of different initiatives and policies that may influence the uptake of biosimilars in different European countries. Recommendations will be formulated on how to create sustainable uptake.

**Methods:**

An overview of policies on biosimilars was obtained via a questionnaire, supplemented with relevant articles. Topics were organized in five themes: availability, pricing, reimbursement, demand-side policies, and recommendations to enhance uptake.

**Results:**

In all countries studied, biological medicines are available. Restrictions are mainly dependent on local organization of the healthcare system. Countries are willing to include biosimilars for reimbursement, but for commercial reasons they are not always marketed. In two thirds of countries, originator and biosimilar products may be subjected to internal reference pricing systems. Few countries have implemented specific incentives targeting physicians. Several countries are implementing pharmacist substitution; however, the scope and rules governing such substitution tend to vary between these countries. Reported educational policies tend to target primarily physicians, whereas fewer initiatives were reported for patients. Recommendations as proposed by the different country experts ranged from the need for information and communication on biosimilars to competitive pricing, more support for switching and guidance on substitution.

**Conclusions:**

Most countries have put in place specific supply-side policies for promoting access to biosimilars. To supplement these measures, we propose that investments should be made to clearly communicate on biosimilars and educate stakeholders. Especially physicians need to be informed on the entry and use of biosimilars in order to create trust. When physicians are well-informed on the treatment options, further incentives should be offered to prescribe biosimilars. Gainsharing can be used as an incentive to prescribe, dispense or use biosimilars. This approach, in combination with binding quota, may support a sustainable biosimilar market.

## Introduction

Biological medicinal products are widely used, e.g., in treatment of autoimmune diseases and cancer, targeting key disease mediators [1]. They are often expensive and continued growth in market share and expenditure is expected in the coming years [2]. This trend puts pressure on already restricted healthcare budgets and may lead to a decrease in patient access to treatment [3]. However, as patents and other exclusivity rights on many high-selling and expensive biological medicinal products are expiring or have expired, non-innovator versions of these biologicals, i.e. biosimilars, may enter the market. Biosimilars are lower cost, but equally effective and safe, alternatives of originator biologicals and may bring relief to pressurized healthcare budgets. In 2016, IMS Health estimated that biosimilars could lead to savings up to €100 billion by 2020 in the United States (US) and the five major countries in the European Union (EU) [4]. In Europe, a legal framework for marketing authorization of biosimilars was established in 2004 [5], and as for all biotechnological products, biosimilars receive marketing authorization via the centralised procedure [6]. The EMA defines a biosimilar as *“a biological medicinal product that contains a version of the active substance of an already authorized original biological medicinal product (reference medicinal product) in the European Economic Area (EEA)*. *Similarity to the reference medicinal product in terms of quality characteristics*, *biological activity*, *safety and efficacy based on a comprehensive comparability exercise needs to be established”*.[7] In 2006 the European Medicines Agency (EMA) approved the first biosimilar, Omnitrope^®^ (somatropin). Since 2006, several product-specific biosimilar guidelines were developed by the EMA [8], and 28 biosimilars have been approved for use in the EU as of June 2017 [9].

After granting marketing authorization to a medicinal product at the European level, local implementation is up to the Member States, and consequently each country can formulate its own biosimilar policies. Differences exist in the pricing and reimbursement procedures, levels of education, characteristics of covered population, and incentivization of stakeholders, leading to variations in uptake of biosimilars [10] and divergences in savings from biosimilars use across Europe, and eventually even within the same country [11]. Experiences from different European countries with biosimilar policies may offer useful insights into current and future uptake of biosimilars.

The aim of this article is to provide an overview of the different initiatives and policies that may influence the uptake of biosimilars, undertaken by health authorities in different European countries. These policies may concern pricing, reimbursement, educational initiatives and incentives for physicians, pharmacists and patients. In 2014, European Biopharmaceutical Enterprises (EBE) conducted a survey to examine pricing and reimbursement policies of off-patent biological medicines in European countries [12]. Our study aims to provide an update on the developments in the biosimilar market and to add value to this survey on various aspects. Incentives for physicians, pharmacists and patients are studied, broadening the scope of the analysis. Furthermore, an additional type of correspondents is included, i.e. the national health authorities, who have introduced these pricing, reimbursement or uptake measures. Our study also includes the new generation of biosimilar monoclonal antibodies, which entered the market in 2015 and are now in full expansion. Three recent articles investigated policies for biosimilars in Europe [13–15], each relying on a literature review to determine existing policies. In contrast, our study used close collaboration with national experts to gain insight in the biosimilar market of their country. These recent articles [13–15] emphasized the need for a better understanding of what drives biosimilar uptake and the establishment of a robust policy framework. Therefore, our study aims to further clarify, with a different methodology, which policies exist. Furthermore, based on the survey results, recommendations will be formulated on how to create a sustainable uptake of biosimilars in Europe.

## Methods

An overview of policies on biosimilars used in European countries was obtained via a questionnaire, administered to different country experts, and supplemented with relevant articles. Topics for the questionnaire were derived from previous work on generic medicines [16] and The Belgian Health Care Knowledge Centre (KCE) report 199 on biosimilars [17]. Topics were organized in five themes: the availability of biosimilars, pricing policies, reimbursement policies, demand-side policies (broken down by the 4Es–Education, Engineering, Economics and Enforcement [18]), and recommendations to enhance uptake of biosimilars. Open as well as closed questions were included. The questionnaire was written in English. The questionnaire as updated in August 2016 can be consulted as supporting information (S1 Questionnaire). All molecules for which a biosimilar was marketed in Europe at that time (filgrastim, somatropin, erythropoietin alfa, insulin glargine, follitropin, infliximab and etanercept) were included. This article presents several highlights from this extensive questionnaire. Results were rearranged in three themes: i) availability of biosimilars, ii) national policies, and iii) recommendations from the country experts.

A pilot survey was carried out in two countries (Slovenia, Lithuania) to validate the questions in terms of clarity, after which the refined questionnaire was sent out to experts of different European countries with knowledge of the biosimilar market. These experts were initially contacts within the Piperska Group [19], which is a network of professionals doing research on the rational use of medicines. These contacts were then further supplemented with other contacts in the network of the KU Leuven / Erasmus MC team. Country experts represented regulatory authorities in different European countries, pricing and reimbursement authorities, health insurance companies, health technology assessment (HTA) bodies, procurement agencies, and academia, and were contacted via e-mail or telephone to participate in this study. Initially, one expert per country was contacted. This expert acted as the national contact, responsible for collecting data for the survey, and contacting additional national stakeholders. When responses were not clear, clarification was requested via e-mail or phone calls. This approach was used before when assessing different policies and their impact, as well as debating key issues such as managed entry agreements, personalized medicine and new models to enhance the managed entry of new medicines [20–26]. The data obtained via the questionnaire were supplemented with relevant articles, and, if possible, responses were validated by contacting other experts in the individual countries. Descriptive statistics were used to analyze the data, with frequencies being reported where appropriate, and open questions being examined via qualitative analysis. During critical review of the manuscript, the experts were asked to include data collected until the end of April 2017. Data was collected between November 2015 and May 2017.

## Results

Data were obtained from 24 countries, 20 EU Member States, plus Iceland, Norway, Russia, and Serbia. The 20 EU Member States, and European Economic Area (EEA) countries Iceland and Norway, fall within the scope of the centralized marketing authorization in line with Regulation (EC) No 726/2004 [6]. This contrasts with Russia and Serbia where authorization of biosimilars is the responsibility of the national health authorities. At the time the study was carried out, biosimilars for filgrastim, somatropin, erythropoietin alfa, insulin glargine, follitropin, infliximab and etanercept had been launched in the European market. In the following paragraphs, results for the different themes in the questionnaire are presented: i) availability of biosimilars throughout Europe, ii) national policies for biosimilars, subdivided in pricing, reimbursement and demand-side policies, and iii) recommendations to enhance uptake of biosimilars as proposed by the different country experts.

### i) Availability of biosimilars

Although biosimilars are approved via the EMA and thereby receive marketing authorization in the European Union, availability of biosimilars can differ across countries. Availability in our study is defined as marketed by the marketing authorization holder in a specific country, but not necessarily funded. As can be seen in S1 Table, biologicals, including biosimilars, of the aforementioned active substances are mainly available in the hospital setting, although most countries also make them available in ambulatory care. In all countries studied, biological medicines are available. Restrictions are mainly dependent on local organization of the healthcare system. Insulin is a product that is typically dispensed via ambulatory care and additionally via hospitals, while infliximab is mainly a hospital product.

Since biologicals are often expensive, funding is necessary to support patient access to these treatments. S2 Table shows which biosimilars are funded in the selected countries, as of April 2017. Whether a specific product is available in a given country, in most cases is a commercial decision of the marketing authorization holder and not of a regulatory or reimbursement agency.

For filgrastim, seven biosimilars are currently authorized, which are marketed by five different companies [9]. As can be seen in S2 Table, the biosimilars of filgrastim that are funded in most countries are, in decreasing order, Zarzio^®^, Nivestim^®^, Tevagrastim^®^, and Accofil^®^. When an epoetin alfa biosimilar is funded in a country, Binocrit^®^ or Retacrit^®^ is chosen. In some countries, no biosimilar for epoetin is funded. This is the case in Estonia, Iceland, Malta, and Russia. In all countries, except Malta, biosimilar somatropin, Omnitrope^®^, is funded (which is the only biosimilar of somatropin that is authorized). In 18 out of 24 countries, Abasaglar^®^, biosimilar insulin glargine, is funded. On the biosimilar follitropin market, Bemfola^®^ or Ovaleap^®^ is funded in all countries, except for Austria, Malta, Russia, and Serbia. With respect to infliximab biosimilars, in almost all countries, Inflectra^®^ as well as Remsima^®^ are funded. Five countries have Flixabi^®^ on the market, which received approval from the EMA in May 2016. Benepali^®^ (biosimilar etanercept) was approved by the EMA in January 2016 and is funded in 14 of the 24 countries.

Germany was the only country in which all registered biosimilars are available and funded. It should be noted that Russia has a different regulatory system from that of the EU, and has many more non-innovator versions of biologicals than indicated in S2 Table. For comparison, S2 Table, only includes EMA approved biosimilars. Also other countries reported the use of non-originator products and next-generation biologicals. For example, in Iceland Neorecormon^®^ (epoetin beta) is commonly used, but no biosimilars of epoetin are funded. In Malta no biosimilars of epoetin are funded (they are not supplied), but Eporatio^®^ (epoetin theta) is.

### ii) National policies

#### Pricing

In all countries, prices of medicinal products used in ambulatory care are regulated by national authorities, although differences in scope and extent of involvement exist. Table 1 provides an overview of biosimilar pricing mechanisms for the different countries. The most frequent biosimilar pricing mechanisms in ambulatory care are pricing a percentage below the price of the originator medicinal product, and the use of a maximum price. The percentage can be fixed or a range subject to negotiation. Maximum prices are often set by external reference pricing, as is the case in Bulgaria, Czech Republic, Iceland, Malta, Latvia, Serbia, and Slovenia. Other mechanisms can also be used to determine the price of the biosimilar for ambulatory care, including free pricing (e.g., Germany, UK), free pricing without exceeding the price of the reference product (e.g., Norway), market forces (e.g., Russia), national tendering (e.g., Malta, Serbia), HTA (e.g., Sweden), and officially same price as reference product (e.g., the Netherlands). In most countries, no single pricing mechanism is used to determine the price of the biosimilar in ambulatory care, but multiple pricing mechanisms are combined.

**Table 1:** Policies for biosimilars in different countries in Europe (April 2017).

Country	Biosimilar pricing in ambulatory care	Internal reference pricing	Incentives to prescribe	Substitution
**Austria**	First biosimilar: -38% on reference product. Second biosimilar: -15% on first biosimilar. Third biosimilar: -10% on second biosimilar. The reference product has to lower its price by 30% three months after the first biosimilar’s entry into reimbursement. After the third biosimilar has entered into reimbursement, the reference product has to match the price of the cheapest available biosimilar in the Code of Reimbursement. All subsequent entries need to offer a rebate of €0.10 on the cheapest alternative. The requested percentage discount for entry into reimbursement of the first biosimilar of a given reference product was decreased in March 2017 making it easier for biosimilars to be included in the Code of Reimbursement, while the mandatory discounts for generic follower medicines were slightly increased. Before that legal reform, both groups of follower medicines had been treated equally.	Yes	Yes	No
**Belgium**	The price of the biosimilar is negotiated on a case per case base, where the maximum price in order to be reimbursed may not exceed the price of the reference product (class 2 reimbursement). Obligatory price reduction for the reference product when the biosimilar enters the market.	No	Yes	No
**Bulgaria**	The manufacturers’ price of the biosimilar cannot be higher than the lowest price of the same medicine in the reference countries for Bulgaria: Romania, France, Latvia, Greece, Slovak republic, Lithuania, Portugal, Italy, Slovenia, Spain, Belgium, Czech republic, Poland, Hungary, Denmark, Finland, Estonia. Then a regressive margins scale at 3 levels exists and ceiling retail price is calculated and published officially.	Yes	No	No
**Croatia**	The price of the biosimilar is determined via external reference pricing (Italy, Slovenia, Czech Republic, Spain, and France). First biosimilar: -15% on reference product. Next biosimilars: -10% on previous biosimilar.	Yes	No	No
**Czech Republic**	Amendment to Act No. 48/1997 Coll., on Public Health Insurance (1st April 2017): The price and reimbursement of the first biosimilar is cut down by 30% (previously 15%) of the price of the reference product. The statutory price of the reference product remains the same, but the reimbursement level is lowered to the price of the biosimilar. The maximum price of the biosimilar is determined via external reference pricing: All EU countries except for Bulgaria, Czech Republic, Estonia, Luxembourg, Germany, Austria, Romania, Cyprus, and Malta.	Yes	No	No
**England (UK)**	Regulated market, but free pricing by the company. Pharmaceutical price regulation scheme (PPRS): a government negotiated process for branded medicines (4–5 years) with The Association of the British Pharmaceutical Industry (ABPI). The latest PPRS negotiations have volume based pricing scheme. If total amount (£) of medicines sold is above the threshold then the government gets a rebate. Biosimilars go predominantly via hospitals, therefore primary care price or the NHS National tariff is less relevant.	No	Yes	No
**Estonia**	The price of the biosimilar is negotiated. For ambulatory use the price has to be at least 15% below the price of the reference product. For hospital use, there is no fixed percentage.	Yes	Yes	Yes
**Finland**	The price of the biosimilar must be below the price of the reference product. The wholesale price of the first reimbursable biosimilar must be at least 30% lower than the approved wholesale price of the reference product. Once the biosimilar is launched, the price of the originator will be re-examined.	No	Yes	No
**France**	Prices are fixed upon negotiation between pharmaceutical companies and the Economic Committee for Medicinal Products (CEPS), typically 10–20% below the price of the reference product, taking into account a range of factors including the drug’s improvement in medical benefit (ASMR) rating versus therapeutic equivalents (Biosimilars are given an ASMR rating of V), the price of the drug in the rest of Europe, and sales volume forecasts.	Yes	Yes	Yes
**Germany**	The price of the biosimilar is freely set by the company. Discounts may be negotiated through tenders by individual healthcare funds.	Yes	Yes	Yes/No
**Iceland**	The price of the biosimilar must not be higher than the lowest wholesale price of four countries: Denmark, Norway, Sweden, and Finland. When the biosimilar is on the market, the price of the reference product shall be reduced to 80% of the original ex-factory price.	No	Yes	No
**Ireland**	The price of the biosimilar is negotiated, typically 10–20% below the price of the reference product.	No	No	No
**Italy**	In general, biosimilars are priced approximately 20% below the price of the reference product, with external reference pricing being used as supporting information for pricing reimbursed medicines.	No	Yes, in some regions	No
**Latvia**	Biosimilar drugs are evaluated by general principles applied to generics: First biosimilar: at least -30% on reference product. Second and third biosimilar: at least -10% on first/second biosimilar Next biosimilars: -5% further decrease The price may not be higher than the third lowest price in Czech Republic, Romania, Slovakia, Hungary, and Denmark, and not higher than the price in Estonia and Lithuania.	Yes	No	Yes
**Malta**	A maximum price is set for national procurement and this maximum is set through reference pricing (based on a basket of countries). The Centralised Supplies Unit procures medicines by tendering. The specifications for procurement are by INN and do not specify a brand name, allowing biosimilars and originator products to compete in the same procurement procedure.	No	No	No
**Netherlands**	The price of the biosimilar is officially the same as the price of the reference product.	Yes	No	No
**Norway**	The price of the biosimilar cannot be higher than the price of the reference product. The vendor decides the price below this level.	No	Yes	No
**Poland**	First biosimilar: -25% on reference product. Second biosimilar: Must be cheaper than the first. Limit groups exist where the cheapest is the limit for the whole group. Data from EU and EFTA have to be presented.	Yes	No	Yes
**Portugal**	External reference pricing, with change in reference countries each year (2017: Spain, France and Italy), to establish the first maximum price. To be reimbursed, the price of the biosimilar must be at least 20% lower than the price of the reference product.	No	Yes, in hospitals	No
**Russia**	90% of the price of the ‘reference product’ (first in Russia) for reimbursement. Market forces determine the price of the biosimilar when not reimbursed.	Yes	No	Yes
**Serbia**	First biosimilar: -30% on reference product, setting the reimbursement rate. Second biosimilar: -10% on first biosimilar. Third biosimilar: -10% on second biosimilar. With a maximum of 90% of the average price of the corresponding biosimilar in Slovenia, Croatia, and Italy. National tendering by brand name can occur.	Yes	No	No
**Slovenia**	If the same biosimilar medicinal product is on the market in one of the reference countries for Slovenia or in any other EU/EEA country, the price is respectively 92% of the lowest price of the biosimilar in the reference countries (Austria, Germany and France) or 92% of a median price in other EU/EEAC countries. If the biosimilar is not on the market in any of the reference countries or EU/EEAC countries, 68% of the price of the reference product is the price of the biosimilar. JAZMP determines maximum allowed price of all medicinal products (in exceptional cases also exceptional higher allowed price), including biosimilars. At the level of pricing, no negotiations take place. Prices are negotiated by the reimbursement body, individual hospitals and in public procurement.	Yes	No	No
**Spain**	The price of the biosimilar is negotiated, typically 25–30% below the price of the reference product. A maximum price is set as a reference price for national procurement.	Yes	Yes, in some regions	No
**Sweden**	HTA, a cost effectiveness analysis, is the base for decisions on pricing of a biosimilar. The price of the biosimilar needs to be the same or lower than the price of the reference product.	No	Yes, regional	No

ASMR: Amélioration du Service Médical Rendu, CEPS: Comité économique des produits de santé (Economic Committee for Medicinal Products), EU: European Union, EFTA: European Free Trade Association, EEA: European Economic Area, HTA: Health technology assessment, INN: International nonproprietary name, JAZMP: Javna agencija Republike Slovenije za zdravila in medicinske pripomočke (Slovenian Agency for medicinal products and medical devices), NHS: National Health Service

For medicinal products used in the hospital setting, tenders occur in all countries, either at a national level or by individual hospitals, and biosimilars may be included. In Belgium, there is a national list price for hospital medicinal products, but the price at hospital level may vary, since hospitals tender individually or in groups. In Malta and Serbia, just as for medicinal products used in ambulatory care, also hospital medicinal products are procured by national tendering. National tendering, organized by the Norwegian Hospital Procurement Trust, Division Pharmaceuticals (*legemiddelinnkjøpssamarbeid*, LIS), also occurs among the hospital group in Norway, where large discounts can be observed (a 69% discount of biosimilar infliximab on the originator medicinal product in 2015, and a 60% discount in 2016) [27]. There is a clear trend towards using international nonproprietary names (INN) in the preparation of tenders. However, tendering by brand name, or other mechanisms (e.g., grouping by indication or therapeutic class) are used as well. The latter is commonly used in Poland, England and France.

#### Reimbursement

In all countries reimbursement is a matter of national authorities. In Italy, England and Russia, also regional budgets can be involved. In England, the decision to reimburse a medicinal product is made by different commissioners depending upon their therapeutic class, e.g., biosimilars of cancer drugs will be reimbursed by National Health Service England and biosimilars of TNF-alpha inhibitors by clinical commissioning groups. In Russia, it is possible for individual regions to create their own programs which supplement the national one.

In the majority of countries, reimbursement is granted for all indications authorized, and not only the indications for which a clinical trial was conducted, as is the case in Croatia and Serbia. In Malta, a protocol exists which specifies the conditions for reimbursement, for example, when a product is not approved as a first-line treatment, it specifies the previous treatment failure before another product can be reimbursed. In Bulgaria, reimbursement in ambulatory care for some biosimilars is based on their International Classification of Diseases (ICD) code [28], while reimbursement for use in the hospital setting is usually granted according to the marketing authorization. This is the case with epoetin, which in ambulatory care is reimbursed only for chronic kidney disease, while in hospitals reimbursement is granted for all authorized indications.

In two thirds of the countries, the originator medicinal product and biosimilar may be subject to internal reference pricing (Table 1). This means a common reimbursement level is set for a group of medicinal products. Internal reference pricing could also contribute to the demand-side, if the biosimilars are presented with lower prices than their reference medicinal products. This is the case in Bulgaria, where physicians are recommended to prescribe biosimilars for treatment-naïve patients.

#### Demand-side policies

Approximately half of the countries have incentives targeting physicians to prescribe biosimilars, as can be seen in Table 1. Within the context of a convention providing specific supplementary remuneration based on attaining public health objectives (rémunération sur objectifs de santé publique, ROSP), a new measure was introduced in France in 2016, which encourages physicians to prescribe at least 20% insulin glargine biosimilars in ambulatory care [29]. Belgium’s ‘Pact of the future’ for the patient with the pharmaceutical industry [30], which aims to ensure patient access to innovate treatments, fosters innovation, and creates a new deontological framework for the pharmaceutical industry, provided the basis for a covenant between the government, the pharmaceutical industry and the medical sector to stimulate the use of biosimilars [30, 31]. In this context, physicians are encouraged to prescribe at least 20% biosimilars for treatment-naïve patients. In Germany, quotas exist for some biosimilars within the context of regionally negotiated economic targets. In addition, there can be incentives for physicians to prescribe economically, e.g., in Austria the physician is called upon to prescribe the most cost-effective product when several therapy options are available, providing an incentive for physicians to prescribe biosimilars. Also in Belgium, biosimilars are part of quotas for prescribing low-cost medicines [32]. Most countries mention budgetary restrictions playing a role in prescribing a biosimilar. In the Netherlands, limitation of prescription of the originator medicinal product once the biosimilar has entered the market is sometimes enforced by insurance companies for reimbursement purposes.

In some countries, physician incentives have been incorporated in pricing and reimbursement mechanisms with a view to stimulate biosimilar uptake. For instance, in Norway, all products paid by the hospitals (also some for outpatient use) are subject to tendering. A ranking is then made by the Norwegian Hospital Procurement Trust, Division Pharmaceuticals (LIS) based on price, and a recommendation is written. The physicians have to follow the ranking and use the cheapest product, which often is a biosimilar, except when there is a clinical reason not to use the cheapest product. With this system, biosimilar infliximab has reached a market share above 95%, and also the market share of biosimilar etanercept has been increasing to above 82%.

Substitution may also influence the uptake of biosimilars. Substitution is defined by the EMA and the European Commission as: *“The practice of dispensing one medicine instead of another equivalent and interchangeable medicine at pharmacy level without consulting the prescriber*.*”* [33] In most countries substitution of biological medicines is not allowed, except for Estonia, France, Latvia, Poland, and Russia (Table 1). In Estonia, medicinal products for ambulatory use have to be prescribed by INN. As a result, substitution concerns treatment-naïve patients as well as patients previously treated with a biological. The pharmacist will inform the patient about the cheapest alternative. Patients can refuse biosimilar substitution, but then have to pay the price difference between the originator medicinal product and the biosimilar out-of-pocket. The physician can prevent substitution through medical justification. In France, legislation allowing substitution of biosimilars has been introduced as part of a new law concerning the social security budget (Art 96 of the 2017 French Social Security Financing Law) and is limited to specific conditions. Substitution is allowed only for treatment-naïve patients initiating treatment (specified by the physician on the prescription) or to ensure continuity with the same biosimilar, if the biosimilar belongs to the same group as the prescribed product (similar biologic group), and if the prescribing physician has not explicitly prohibited substitution. If the pharmacist substitutes the prescribed biological for the biosimilar, they must write down the name of the dispensed product on the prescription and inform the prescribing physician. To date, no implementing decree has been enacted, thus in practice substitution is not yet taking place. Latvia also allows for substitution at the pharmacy level. If a doctor has prescribed the originator medicinal product and has not indicated on the prescription that the prescribed medicine may not be substituted, it is the duty of the pharmacist to inform the patient about the cheapest alternative. Patients can refuse biosimilar substitution, but then have to pay the price difference between the originator medicinal product and the biosimilar. For newly diagnosed patients, INN prescribing has to be used, and then the duty of the pharmacist is to dispense the cheapest reimbursable medicinal product, conforming to the name, pharmaceutical form and strength. The patient cannot choose. In Poland, substitution is allowed by law within reference groups, and the pharmacist should discuss substitution with the patient. In Russia, physicians prescribe by INN, but they can prevent substitution by providing a medical reason. Patients can refuse and explain their reasons to the physician, or they can buy the brand name out-of-pocket. In Germany, subgroups of bioidenticals are defined for some biologicals [34], where pharmacist substitution is allowed unless specifically forbidden by the prescribing physician, the so-called *Aut-idem-Regelung* (i.e., rules regarding same-substance substitution). Bioidenticals are biosimilars to the same reference product and produced by the same manufacturer and manufacturing process, but marketed under different trade names, e.g., Inflectra^®^ and Remsima^®^.

A variety of educational policies are implemented across countries. In most countries, local initiatives exist among physicians in hospitals or ambulatory care. Also prescribing guidelines and clinical guidelines can inform physicians. In some countries, including Portugal and the Netherlands, scientific conferences are organized by, e.g., health authorities, to educate stakeholders and stimulate the use of biosimilars. In Norway, the Norwegian Hospital Procurement Trust, Division Pharmaceuticals (LIS) arranges every year several seminars for hospitals, where results of the tenders are presented. In addition, lectures are organized of which several discussed the topic of biosimilars. Reported educational policies tend to target in the first instance physicians, whereas fewer policies were reported for patients. Patients are mainly informed via patient organizations (e.g., via educational initiatives and surveys) or leaflets (e.g., a patient brochure in Portugal, explaining what a biosimilar is), or via letters when exposed to a switch.

### iii) Recommendations as proposed by the country experts

It was clear for each country that various hurdles exist to the development of the biosimilar market and that the full potential of biosimilars is not yet captured. Possible solutions were suggested to overcome these obstacles, and although some were country-specific, we list here overall recommendations derived from the survey results to support market uptake of biosimilars.

The main hurdle for all countries was limited knowledge of biosimilars among key stakeholders and the lack of education, especially for physicians and patients. It is important that physicians and patients can make an informed decision. There is a clear need for more information and better communication on the use of biosimilars. This information should come from an independent institution and should be precise and reliable. Lessons and trainings could be organized, for example in hospitals, where most biosimilars will be prescribed or at least the therapy is initiated. For patients, a questions and answers (Q&A) document could help to address frequently asked questions. The use of a common platform to share experiences and knowledge was suggested, and also ongoing publication of safety and efficacy data, so that evidence-based decisions can be made. Furthermore, concerns related to switching and substitution possibilities, and the definition of a treatment-naïve patient should be addressed. Introduction of biosimilars in prescribing guidelines and clinical guidelines will be key to ensure increased uptake.

Competitive, sustainable pricing is needed. Some suggested the price difference should be large enough to incentivize physicians or to use obligatory price reductions for the originator medicinal product. National tendering, tendering by INN, and a ‘product of the month’-system could be effective to achieve lower prices. HTA could assist in budget planning, and help incentivize physicians. Pharmaceutical companies and national authorities should collaborate, on a national level as well as between countries, to support affordability through competitive price systems.

Several experts noted that, if switching from the originator medicinal product to the biosimilar is accepted as a principle, then there should be more support for this process. The burden of switching now lies with the prescribing physician, and monitoring of patients on a biosimilar can be very resource intensive. Switching programs can for example be supported via insurance companies, but also pharmaceutical companies could provide supportive infrastructure (e.g., IT infrastructure), which supports pooling of data between different centers. Gain sharing, which is here defined as an incentive where part of the savings from using biosimilars goes to the hospital or prescribing physician, is also suggested as a meaningful solution.

A hurdle that was identified by the country experts is that the EMA, which has all the data on the reference medicinal product, does not decide on biosimilar substitution and derogates this decision to the Member States. The question of interchangeability needs to be addressed at the European level as well. The development of a list for appropriate substitution would help. One of the experts offered mandatory use of the cheapest product, as decided by health authorities, as a measure to assist biosimilar uptake.

Furthermore, experts agreed that measures should be introduced to promote rational prescribing, and that adherence to these measures should be verified. For treatment-naïve patients it is generally accepted that they can safely be started on the biosimilar. Here, quota can help to increase uptake of biosimilars.

The experts find leadership is needed on policies, and for this transparency and trust between the different stakeholders is key. It was proposed by some country experts that payer and insurance companies could be in the right position to lead on prescribing changes. However, biosimilar policies have to include all stakeholders, from patient groups, doctors, hospitals to national institutions responsible for pricing and reimbursement. A multi-stakeholder approach is deemed needed in the exercise leading to a more effective allocation of available resources in view of optimal global health management. A clear strategy and a stratified prioritization exercise for the allocation of resources is needed. HTA can assist in reimbursement decisions.

## Discussion

This article has presented a comprehensive overview of different policies for biosimilars in Europe, and has elicited recommendations from experts to enhance their uptake. Our article has mapped the diversity of biosimilar policies implemented in European countries, and showed that policy makers have adopted different approaches in supporting the biosimilar market.

Our study adds to the scarce literature about biosimilar uptake policies. Although Renwick *et al*. performed a literature review in 2016 on post-market policy considerations for biosimilar oncology drugs [15], our study included all biosimilars available at that point and used a questionnaire and close collaboration with the experts for recent information and insight in their country. A recent quantitative study by Rémuzat *et al*. corroborated the importance of mapping biosimilar uptake policies as they found that biosimilar market penetration is higher when supported by uptake policies [13]. Identification of national policies in this study was done via a literature review [14].

### i) Availability of biosimilars

Our study inquired about the setting in which a product is available. However, S1 Table only shows whether it is possible to receive a specific active substance in ambulatory care or in the hospital setting. Via which setting a product is mainly distributed cannot be determined. Complex, expensive products are often distributed via the hospital, e.g., infliximab.

This study shows which biosimilars are funded for the selected countries. However, funding of a biosimilar is no indication of the actual use of the biosimilar in a country. Recently approved biosimilars, as Inflectra^®^ and Remsima^®^, seem to be well accepted and integrated in the healthcare systems of the different countries. On the one hand, this may suggest there is a high need for lower cost alternatives for these complex and expensive molecules, in contrast to simpler molecules, like insulin, epoetin, and follitropin. On the other hand, this might indicate an increasing trust in the biosimilar pathway. This evolution will become clear in the upcoming years, knowing the pipeline of biosimilars (both monoclonal antibodies as well as smaller biologicals) [35].

### ii) National policies

Our analysis indicated that originator and biosimilar products may be subjected to internal reference pricing systems in two thirds of countries. If these products are included in the same reference group, this implicitly means that the originator product and the biosimilar product are considered to be interchangeable. This is noteworthy because the EMA does not assess interchangeability when considering the marketing authorization of a biosimilar. It can thus be inferred that the in/exclusion of originator and biosimilar products in internal reference pricing is likely to reflect the different positions of European countries towards interchangeability within the context of switching. Starting treatment-naïve patients on a biosimilar is generally accepted as safe, but no consensus exists among countries for switching of existing patients [8].

Approximately half of the countries have implemented specific incentives targeting physicians, such as quota. Quota arrangements require physicians to prescribe a minimum percentage of biosimilars when prescribing a biological medicine. When quotas are in place, it is important that adherence to this quota can be verified, for example, by prescription monitoring. Quotas which are not binding tend to miss their target, as is the case in several regions in Italy, where the individual regions autonomously regulate market access and thus can introduce measures on biosimilar use.

Although most European countries do not allow biosimilar substitution at the pharmacy level, our results showed that several countries are implementing pharmacist substitution, even though the scope and rules governing such substitution tend to vary between these countries. In the study of Reiland *et al*. (an update of the 2014 EBE survey) it is noted that over the last years additional countries are allowing pharmacy-level substitution of biologicals [36]. To date, there is a lack of knowledge about pharmacist biosimilar substitution practices in general, and the impact of various conditions governing substitution in particular. These will be research areas for the future.

Although healthcare professionals and patients appreciate that the pharmaceutical budget is finite and that biosimilars can help to sustain healthcare, their knowledge and experience with biosimilars tends to be limited [37–39]. In response to this, the European Commission has issued Q&A documents about biosimilars targeting healthcare professionals and patients [33, 40, 41], and also individual countries have started initiatives to educate stakeholders and help combat misconceptions and misinformation [42]. Limited literature exists in which clear educational approaches are proposed. Possible ways of increasing awareness are integrating biosimilars in the curriculum of pharmacy students [43], and dissemination of positions statements by scientific or professional societies, for example the European Society for Medical Oncology (ESMO) [44]. Our experts additionally highlighted the need for unbiased information, prescribing guidelines, and training schools for practicing healthcare professionals.

### iii) Recommendations as proposed by the country experts

It was proposed by the country experts to use mandatory price reductions on the originator biological. Although this may lead to savings in the healthcare budget, it may turn into a disincentive for companies developing a biosimilar, by eroding their competitive advantage and limiting their return on investment [14].

As indicated before, the European Commission plays an important role in education of stakeholders and the distribution of information from independent institutions [33, 40, 41], as recommended by the different country experts. An increasing number of publications on the safety and efficacy of biosimilars can be found [45–47], for example results of the Norwegian government funded NOR-SWITCH trial on the safety of switching from originator infliximab to its biosimilar version, CT-P13 [48].

However, a disincentive exists for healthcare providers that switch to the biosimilar, requiring extra time and efforts. This can be solved by a mechanism called gain sharing, i.e. that healthcare providers are rewarded in some way for their efforts to introduce cost savings into the healthcare system.

### Limitations

Our study suffered from several limitations. Our analysis was based on a general questionnaire that allowed us to carry out a comparative analysis of biosimilar policies between countries, but was less adapted to the local policy environment and may not have picked up country-specific information. As a result, there might have been difficulties with interpreting and answering the questions. Additionally, experts might not know about the various initiatives that exist in their country, and local policies. In response to this limitation, open boxes were added to elaborate on each question and after each theme experts were asked if they had additional comments on the questions in that theme. This permitted experts to provide additional country-specific information. Finally, experts reviewed the manuscript to decrease interpretation errors and to add additional information.

Using initially one expert per country could raise questions on the validity of the answers. This was mitigated by supplementing the data with relevant literature, and contact with extra experts. Since the questionnaire was only available in English, this could have potentially excluded suitable experts, although contacted experts have functions which imply that an intermediary to advanced level of English is present.

### Future research

Our study that mapped biosimilar uptake policies can provide a platform for further research that quantifies the impact of specific policies on the biosimilar uptake level, or that contrasts high-uptake countries with low-uptake countries and the relation of biosimilar uptake with the implementation of specific policies.

## Conclusion

This article has mapped biosimilar uptake policies in 24 countries and identified possible approaches that were proposed by local experts to enhance the uptake of biosimilars in Europe. This mapping exercise underlines that countries are implementing a variety of biosimilar uptake policies, highlighting the need for future studies to learn from this experience and to investigate the impact of specific policies on biosimilar uptake levels.

Most countries have put in place specific supply-side policies for promoting access to biosimilars. To supplement these measures, we propose that investments should be made by individual countries, and on a European level, to clearly communicate on biosimilars and educate stakeholders. Especially physicians who prescribe treatments with biological medicinal products need to be informed on the entry and use of biosimilars in order to create trust in these products. When physicians are well-informed on the treatment options, further incentives should be offered to prescribe biosimilars. Gain sharing can be used as an incentive to prescribe, dispense or use biosimilars. This approach, in combination with binding quota, may support a sustainable biosimilar market. It seems the attitude of countries towards substitution of biologicals is changing, with several countries implementing (restricted) pharmacist substitution. This may indicate growing importance of incentives targeting pharmacists to dispense biosimilars.

## Supporting information

S1 Questionnaire Questionnaire biosimilar medicines market–The situation in your country. (DOC)

S1 Table Setting in which biological medicinal products are available in different countries in Europe (April 2017). (DOCX)

S2 Table Brand names of funded biosimilars in different countries in Europe (April 2017). (DOCX)
