# Supplementary material for: Policies for biosimilar uptake in Europe: An overview
Source: PLoS One. 2017 Dec 28;12(12):e0190147. doi: 10.1371/journal.pone.0190147 (PMC5746224; doi:10.1371/journal.pone.0190147)
Supplement: S1 Questionnaire — (DOC) [file pone.0190147.s001.doc]

**Questionnaire biosimilar medicines market –**

**The situation in your country**

We would appreciate it if you would complete the questionnaire **electronically**.

**Instructions for the country coordinators:**

This study aims to create an overview of different policies utilised among different health authorities in European countries to enhance the market access of biosimilars. Furthermore, based on the results of the questionnaire, we aim to make policy recommendations to facilitate uptake of biosimilars. Therefore we need the help of biosimilar experts from different European countries.

This questionnaire refers to data and policies for *biosimilar medicines* in your country for the most recent years (unless indicated otherwise)*.*

The *questionnaire* consists of *three different parts*. In the first part we wish to assess the current situation of the biosimilar medicines market and general legislation. In the second part we wish to assess specific policies which affect biosimilar uptake. These policies are collated under the 4 Es: Economics, Engineering, Education and Enforcement (Economics - Includes financial incentives for physicians, patients or pharmacists; Engineering - Refers to organisational or managerial interventions, e.g. prescribing targets and therapeutic switching initiatives; Education - Activities range from printed guidelines to more intensive strategies, including academic detailing and benchmarking physician prescribing habits; Enforcement – Includes regulations by law such as prescribing restrictions and compulsory biosimilar substitution). Finally, in the third part we would like to know your opinion on potential ways to further develop the biosimilar medicines market in a sustainable way.

It would be appreciated if you could answer all the questions by yourself or pertinent colleagues within your country. However, if there are questions which are unclear or where the answer is unknown, you can leave these questions blank. Please answer the best representation of your country if regional differences exist.

Information gathered in this questionnaire will be utilized in the context of a scientific research project undertaken by KU Leuven, and the Piperska network. Your name and function will be made available in a general acknowledgement in order to provide a pertinent source of our information, as well as be included as a co-author in any subsequent peer-reviewed publications if wished. If you would like to remain anonymous you may note this in the space provided under ‘contact details’. We would still like you to fill out the contact details for own internal use.

Please respond to questions electronically. You can move forward through the main body of the questionnaire by pressing "Tab" and backwards by pressing "Shift + Tab", or you can use the scroll feature and the mouse. Boxes can be ticked and ticks can be removed by double-clicking the mouse.

**If you have any questions, do not hesitate to contact me at the correspondence address.***(****Evelien Moorkens*** *-* [*evelien.moorkens@kuleuven.be*](mailto:evelien.moorkens@kuleuven.be)*)*

CONTACT DETAILS FOR COORDINATING PERSON COMPLETING THE FORM

| Name (First + last): |  | I would like to remain anonymous |
| --- | --- | --- |
| Function: |  | |
| Institution: |  | |
| Type of institution: |  | |
| Unit: |  | |
| Address (work): |  | |
| E-mail (work): |  | |
| Tel.: |  | |
| Country: |  | |

| **Part 1: Current situation (2015-2016)** | | | | | | | | | | | | | | |
| --- | --- | --- | --- | --- | --- | --- | --- | --- | --- | --- | --- | --- | --- | --- |
| **General** | | | | | | | | | | | | | | |
|  | Please indicate which of the following biosimilar medicines are available and are reimbursed/funded in your country.  *(More than one box can be ticked.)* | | | | | | | | | | | | | |
|  | Filgrastim | Erythropoietin | | | Somatropin | Infliximab | | Insulin | | | | Follitropin | | |
|  | Accofil  Biograstim   Filgrastim Hexal  Grastofil  Nivestim  Ratiograstim  Tevagrastim  Zarzio | Abseamed  Binocrit  Epoetin alfa Hexal  Retacrit  Silapo | | | Omnitrope | Inflectra  Remsima  Flixabi | | Abasaglar | | | | Bemfola  Ovaleap | | |
|  | Etanercept |  | | |  |  | |  | | | |  | | |
|  | Benepali |  | | |  |  | |  | | | |  | | |
|  | Please indicate in which setting the following biological medicines are available.  *(More than one box can be ticked.)* | | | | | | | | | | | | | |
|  | Ambulatory care Hospitals  Filgrastim  Somatropin  Erythropoietin  Infliximab  Insulin  Follitropin  Etanercept | | | | | | | | | | | | | |
|  | Are there any additional comments relating to the general situation regarding the availability of biologicals/biosimilars in your country? | | | | | | | | | | | | | |
|  |  |  |  |  |  |  |  |  |  |  |  |  |  |  |
| **Pricing** | | | | | | | | | | | | | | |
|  | If applicable, is price regulation of medicines in ambulatory care (out-of-hospital) a matter of regional or national authorities in your country? | | | | | | | | | | | | | |
|  | Regional | | | | | National | | | | | | | | |
|  | Are prices of biosimilar medicines in ambulatory care regulated, or established through market forces? | | | | | | | | | | | | | |
|  | Regulated | | | | | Established through market forces (e.g. Tendering (public procurement)) | | | | | | | | |
|  | If regulated, please specify how: | | | | | | | | | | | | | |
|  | The average of selected EU countries (countries:      ) | | | | | | | | | | | | | |
|  | Percentage below the original biological medicine (      % ) | | | | | | | | | | | | | |
|  | Maximum price | | | | | | | | | | | | | |
|  | Negotiable | | | | | | | | | | | | | |
|  | Other: | | | | | | | | | | | | | |
|  | Please elaborate: | | | | | | | | | | | | | |
|  | Are prices of biosimilar medicines in hospitals regulated on a national level in your country? | | | | | | | | | | | | | |
|  | Yes | | | | | No | | | | | | | | |
|  | If yes, how is this undertaken? | | | | | | | | | | | | | |
|  | If no, what mechanisms are in place to determine prices of biosimilars in hospitals? | | | | | | | | | | | | | |
|  | Is tendering (public procurement) a practice which occurs in hospitals in your country? | | | | | | | | | | | | | |
|  | Yes | | | | | No | | | | | | | | |
|  | If yes, are biosimilars part of this process? | | | | | Yes | | No | | | | | | |
|  | To which biosimilars does this currently apply? | | | | | | | | | | | | | |
|  | Filgrastim | Somatropin | Erythropoietin | | | Infliximab | | Insulin | | | | Follitropin | | Etanercept |
|  | How are tenders prepared? | | | | | | | | | | | | | |
|  | By International Nonproprietary Name (INN) | | | | | | | | | | | | | |
|  | By brand name | | | | | | | | | | | | | |
|  | Other | | | | | | | | | | | | | |
|  | Please elaborate: | | | | | | | | | | | | | |
|  | Are prices that hospitals pay for medicines transparent (or are discounts/rebates confidential)? | | | | | | | | | | | | | |
|  | Yes | | | | | | No | | | | | | | |
|  | Please elaborate: | | | | | | | | | | | | | |
|  | Are there any additional comments relating to factors which influence the pricing of biosimilar medicines? | | | | | | | | | | | | | |
|  |  |  |  |  |  |  |  |  |  |  |  |  |  |  |
| **Reimbursement** | | | | | | | | | | | | | | |
| *Complete this section if relevant to your country/region.* | | | | | | | | | | | | | | |
|  | Is reimbursement a matter of regional or national authorities in your country? | | | | | | | | | | | | | |
|  | Regional | | | | | National | | | | | | | | |
|  | Is biosimilar reimbursement granted to the indication for which a clinical trial has been conducted only, or all indications (i.e. the indication for which a clinical trial has been conducted and all extrapolated indications)? | | | | | | | | | | | | | |
|  | The indication for which a clinical trial has been conducted only | | | | | All indications | | | | | | | Other (e.g., reimbursement differs between insurance companies) | |
|  | Please elaborate: | | | | | | | | | | | | | |
|  | Are original biological medicines or biosimilar medicines included in a reference pricing system in your country? | | | | | | | | | | | | | |
|  | Biosimilars  Yes  No | | | | | Original biologicals  Yes  No | | | | | | | | |
|  | If yes, which products does this concern? | | | | | | | | | | | | | |
|  | Please elaborate: | | | | | | | | | | | | | |
|  | Are there any additional comments relating to factors which influence the reimbursement of biosimilar medicines? If so, what are these? | | | | | | | | | | | | | |
|  |  |  |  |  |  |  |  |  |  |  |  |  |  |  |
| **Part 2: Demand-side policies regarding uptake (collated under the 4 Es)** | | | | | | | | | | | | | | |
| **Economics** | | | | | | | | | | | | | | |
|  | Are there any incentives for physicians to prescribe biosimilar medicines in your country? | | | | | | | | | | | | | |
|  | Yes | | | | | No | | | | | | | | |
| ***If no, please go on to question 18.*** | | | | | | | | | | | | | | |
|  | Are there any financial incentives for physicians to prescribe biosimilar medicines? Please also specify whether they apply to ambulatory care, hospital physicians or both. | | | | | | | | | | | | | |
|  | Yes, budgetary restrictions  Please specify: | | | | | Ambulatory care | | | | | | Hospital | | |
|  | Yes, budgetary incentives  Please specify: | | | | | Ambulatory care | | | | | | Hospital | | |
|  | Yes, other  Please specify: | | | | | Ambulatory care | | | | | | Hospital | | |
|  | No | | | | | | | | | | | | | |
|  | Do incentives for physicians (e.g. guidelines, quotas, etc.) differentiate between treatment naïve patients and existing patients? | | | | | | | | | | | | | |
|  | Yes | | | | | No | | | | | | | | |
|  | Please elaborate: | | | | | | | | | | | | | |
|  | Are pharmacists financially penalized for dispensing biosimilar medicines? | | | | | | | | | | | | | |
|  | Yes | | | | | No | | | | | | | | |
|  | If yes, please specify how: | | | | | | | | | | | | | |
|  | Is there any patient co-payment for biological medicines? | | | | | | | | | | | | | |
|  | Yes | | | | | No | | | | | | | | |
|  | If yes, please specify which molecules and what it’s based on: | | | | | | | | | | | | | |
|  | Is there a difference between the co-payment for original biological and biosimilar medicines? | | | | | | | | | | | | | |
|  | Yes | | | | | No | | | | | | | | |
|  | If yes, please specify: | | | | | | | | | | | | | |
|  | Are there any additional comments relating to economic measures? | | | | | | | | | | | | | |
|  |  |  |  |  |  |  |  |  |  |  |  |  |  |  |
| **Engineering** | | | | | | | | | | | | | | |
|  | Do physicians need to prescribe a certain percentage of biosimilar medicines (prescription quota)? | | | | | | | | | | | | | |
|  | Yes | | | | | No | | | | | | | | |
|  | If yes, please also specify whether this applies to treatment naïve patients, switching or both. | | | | | | | | | | | | | |
|  | Treatment naïve patients | | | Switching | | | | | | Both | | | | |
|  | Please elaborate: | | | | | | | | | | | | | |
|  | If yes, please also specify whether this applies to ambulatory care, hospital physicians or both. | | | | | | | | | | | | | |
|  | Ambulatory care | | | Hospital | | | | | | Both | | | | |
|  | Please elaborate: | | | | | | | | | | | | | |
|  | Is biosimilar substitution in your country legally allowed?  (Biosimilar substitution means the pharmacist substituting the prescribed original biological medicine with a biosimilar medicine without consultation of the prescriber.) | | | | | | | | | | | | | |
|  | Yes | | | | | No | | | | | | | | |
|  | If no, how is this regulated? | | | | | | | | | | | | | |
|  | By law | | By guidelines | | | | | | Other: | | | | | |
|  | If yes, what is the legal basis? | | | | | | | | | | | | | |
| ***If substitution is not allowed, please go on to question 30.*** | | | | | | | | | | | | | | |
|  | For which kind of patients is substitution allowed? | | | | | | | | | | | | | |
|  | Treatment naive patients | | Existing patients | | | | | | Both | | | | | |
|  | For which biosimilar medicines is substitution allowed | | | | | | | | | | | | | |
|  | All biosimilars | | | | | Biosimilars on a limited list | | | | | | | | |
|  | Please specify which products are allowed to be substituted: | | | | | | | | | | | | | |
|  | Do physicians explicitly need to give permission for biosimilar substitution? | | | | | | | | | | | | | |
|  | Yes | | | | | No | | | | | | | | |
|  | If yes, please specify how: | | | | | | | | | | | | | |
|  | Can physicians prevent biosimilar substitution? | | | | | | | | | | | | | |
|  | Yes | | | | | No | | | | | Not applicable | | | |
|  | If yes, please specify how: | | | | | | | | | | | | | |
|  | Can patients refuse biosimilar substitution? | | | | | | | | | | | | | |
|  | Yes | | | | | No | | | | | | | | |
|  | If yes, please specify: | | | | | | | | | | | | | |
|  | Are pharmacists legally obliged to inform patients of substitution | | | | | | | | | | | | | |
|  | Yes | | | | | No | | | | | | | | |
|  | Are there any additional comments relating to engineering measures? | | | | | | | | | | | | | |
|  |  |  |  |  |  |  |  |  |  |  |  |  |  |  |
| **Education** | | | | | | | | | | | | | | |
|  | Are there initiatives in place to educate physicians regarding biosimilar medicines and/or assist physicians to prescribe biosimilar medicines? Please also specify whether this applies to ambulatory care, hospital physicians or both. | | | | | | | | | | | | | |
|  | Yes | | | | | No | | | | | | | | |
|  | If yes, in which way?  *(Multiple answers possible. Please tick all relevant boxes.)* | | | | | | | | | | | | | |
|  | By educational initiatives among physicians | | | | | Ambulatory care | | | | | | Hospital | | |
|  | By prescribing guidelines | | | | | Ambulatory care | | | | | | Hospital | | |
|  | By prescription audits (i.e. regulator monitors physicians’ prescribing profiles and provides them with feedback) | | | | | Ambulatory care | | | | | | Hospital | | |
|  | By electronic prescribing | | | | | Ambulatory care | | | | | | Hospital | | |
|  | Other way  Please specify: | | | | | Ambulatory care | | | | | | Hospital | | |
|  | Please elaborate: | | | | | | | | | | | | | |
|  | Have there been informational campaigns targeting physicians to stimulate the use of biosimilars? | | | | | | | | | | | | | |
|  | Yes | | | | | No | | | | | | | | |
|  | If yes, please elaborate: | | | | | | | | | | | | | |
|  | Have there been information campaigns to inform patients about biosimilar medicines? | | | | | | | | | | | | | |
|  | Yes | | | | | No | | | | | | | | |
|  | If yes, please specify: | | | | | | | | | | | | | |
|  | TV campaign | | Leaflets | | | | | | Advertising | | | | | |
|  | Radio | | | | | Websites | | | | | | | | |
|  | Other: | | | | | | | | | | | | | |
|  | Who was the sponsor of these information campaigns?  *(More than one box can be ticked.)* | | | | | | | | | | | | | |
|  | Health authorities and/or (national / local) government | | | | | | | | | | | | | |
|  | Biosimilar companies | | | | | | | | | | | | | |
|  | Original biological companies | | | | | | | | | | | | | |
|  | Consumer organisations | | | | | | | | | | | | | |
|  | Other: | | | | | | | | | | | | | |
|  | Are there any additional comments relating to educational measures? | | | | | | | | | | | | | |
|  |  |  |  |  |  |  |  |  |  |  |  |  |  |  |
| **Enforcement** | | | | | | | | | | | | | | |
|  | Are there any programmes to limit the prescribing of the originator once biosimilar medicines are in place, e.g. prescribing restrictions? | | | | | | | | | | | | | |
|  | Yes | | | | | No | | | | | | | | |
|  | If yes, please elaborate, as well as provide information on how prescribing restrictions are monitored in practice. | | | | | | | | | | | | | |
|  | Are there any additional comments relating to enforcement measures? | | | | | | | | | | | | | |
|  |  |  |  |  |  |  |  |  |  |  |  |  |  |  |
| **Part 3: Possible solutions** | | | | | | | | | | | | | | |
|  | Based on your previous answers, what policies could be implemented to assist biosimilar uptake in your country? | | | | | | | | | | | | | |
|  | **Relating to pricing:** | | | | | | | | | | | | | |
|  | **Relating to reimbursement:** | | | | | | | | | | | | | |
|  | **Relating to economics:** | | | | | | | | | | | | | |
|  | **Relating to engineering:** | | | | | | | | | | | | | |
|  | **Relating to education:** | | | | | | | | | | | | | |
|  | **Relating to enforcement:** | | | | | | | | | | | | | |

**THANK YOU FOR YOUR CO-OPERATION**

**PLEASE RETURN THE QUESTIONNAIRE ELECTRONICALLY TO:**

**Correspondence address:**

**Evelien Moorkens** - [evelien.moorkens@kuleuven.be](mailto:evelien.moorkens@kuleuven.be)
