# Supplementary material for: Policies for biosimilar uptake in Europe: An overview
Source: PLoS One. 2017 Dec 28;12(12):e0190147. doi: 10.1371/journal.pone.0190147 (PMC5746224; doi:10.1371/journal.pone.0190147)
Supplement: S1 Table — (DOCX) [file pone.0190147.s002.docx]

**S1 Table: Setting in which biological medicinal products are available in different countries in Europe (April 2017)**

| Country | Filgrastim | | Epoetin | | Somatropin | | Insulin | | Follitropin | | Infliximab | | Etanercept | |
| --- | --- | --- | --- | --- | --- | --- | --- | --- | --- | --- | --- | --- | --- | --- |
|  | AC | H | AC | H | AC | H | AC | H | AC | H | AC | H | AC | H |
| EU countries | | | | | | | | | | | | | | |
| Austria | x | x | x | x | x | x | x | x | x | x | x | x | x | x |
| Belgium | x | x | - | x | x | x | x | x | x | x | - | x | x | x |
| Bulgaria | - | x | x | x | x | x | x | x | - | x | x | x | x | x |
| Croatia | - | x | - | x | - | x | x | x | - | x | - | x | - | x |
| Czech Republic | x | x | x | x | x | x | x | x | x | x | x | x | x | x |
| England (UK) | - | x | x | x | x | x | x | x | x | x | - | x | - | x |
| Estonia | - | x | x | x | x | - | x | x | x | x | - | x | - | x |
| Finland | x | x | x | x | x | x | x | x | x | x | x | x | x | x |
| France | x | x | x | x | x | x | x | x | x | x | - | x | x | x |
| Germany | x | x | x | x | x | x | x | x | x | x | x | x | x | x |
| Ireland | x | x | x | x | x | x | x | x | x | x | - | x | x | x |
| Italy | x | x | x | x | x | x | x | x | x | x | - | x | x | x |
| Latvia | x | x | x | x | x | - | x | x | x | - | x | - | x | - |
| Malta | x | x | x | x | x | x | x | x | x | x | - | x | x | x |
| Netherlands | x | x | x | x | - | x | x | x | - | x | - | x | - | x |
| Poland | x | x | - | x | - | x | x | - | x | - | - | x | - | x |
| Portugal | - | x | - | x | - | x | x | - | x | - | - | x | - | x |
| Slovenia | x | x | x | x | - | x | x | x | x | x | x | x | x | x |
| Spain | - | x | - | x | - | x | x | - | x | - | - | x | - | x |
| Sweden | x | x | x | x | x | - | x | - | x | - | - | x | x | - |
| Non-EU countries | | | | | | | | | | | | | | |
| Iceland | x | x | x | x | x | x | x | x | x | x | - | x | x | x |
| Norway | x | x | x | x | x | x | x | x | x | x | - | x | x | x |
| Russia | - | x | - | x | x | x | x | x | - | x | - | x | x | x |
| Serbia | - | x | - | x | x | - | x | x | - | x | - | x | - | x |
| AC = Ambulatory care, H = Hospital | | | | | | | | | | | | | | |
