# Supplementary material for: Policies for biosimilar uptake in Europe: An overview
Source: PLoS One. 2017 Dec 28;12(12):e0190147. doi: 10.1371/journal.pone.0190147 (PMC5746224; doi:10.1371/journal.pone.0190147)
Supplement: S2 Table — (DOCX) [file pone.0190147.s003.docx]

**S2 Table: Brand names of funded biosimilars in different countries in Europe (April 2017)**

| Country | Filgrastim | Epoetin | Somatropin | Insulin | Follitropin | Infliximab | Etanercept |
| --- | --- | --- | --- | --- | --- | --- | --- |
| EU countries | | | | | | | |
| Austria | - Accofil^®^ - Nivestim^®^ - Ratiograstim^®^ - Zarzio^®^ | - Abseamed^®^ - Binocrit^®^ - Retacrit^®^ | - Omnitrope^®^ | - | - | (Inflectra^®^ under review; Remsima^®^ under review) | (Benepali^®^ under review) |
| Belgium | - Accofil^®^ - Nivestim^®^ - Tevagrastim^®^ - Zarzio^®^ | - Binocrit^®^ - Retacrit^®^ | - Omnitrope^®^ | - Abasaglar^®^ | - Bemfola^®^ - Ovaleap^®^ | - Inflectra^®^ - Remsima^®^ | - Benepali^®^ |
| Bulgaria | - Nivestim^®^ - Zarzio^®^ | - Binocrit^®^ - Retacrit^®^ | - Omnitrope^®^ | - Abasaglar^®^ | - Bemfola^®^ | - Inflectra^®^ - Remsima^®^ | - |
| Croatia | - Accofil^®^ - Grastofil^®^ - Nivestim^®^ - Tevagrastim^®^ - Zarzio^®^ | - Binocrit^®^ - Retacrit^®^ | - Omnitrope^®^ | - Abasaglar^®^ | - Ovaleap^®^ - Bemfola^®^ | - Inflectra^®^ - Remsima^®^ | - |
| Czech Republic | - Accofil^®^ - Nivestim^®^ - Tevagrastim^®^ - Zarzio^®^ | - Binocrit^®^ - Retacrit^®^ | - Omnitrope^®^ | - Abasaglar^®^ | - Ovaleap^®^ - Bemfola^®^ | - Inflectra^®^ - Remsima^®^ - Flixabi^®^ | - Benepali^®^ |
| England (UK) | - Nivestim^®^ - Ratiograstim^®^ - Tevagrastim^®^ - Zarzio^®^ | - Binocrit^®^ - Retacrit^®^ | - Omnitrope^®^ | - Abasaglar^®^ | - Bemfola^®^ | - Inflectra^®^ - Remsima^®^ | - Benepali^®^ |
| Estonia | - Nivestim^®^ - Tevagrastim^®^ - Zarzio^®^ | - | - Omnitrope^®^ | - Abasaglar^®^ | - Bemfola^®^ - Ovaleap^®^ | - Inflectra^®^ - Remsima^®^ | - |
| Finland | - Accofil^®^ - Nivestim^®^ - Ratiograstim^®^ - Zarzio^®^ | - Binocrit^®^ - Retacrit^®^ | - Omnitrope^®^ | - Abasaglar^®^ | - Bemfola^®^ | - Inflectra^®^ - Remsima^®^ | - |
| France | - Accofil^®^ - Nivestim^®^ - Ratiograstim^®^ - Tevagrastim^®^ - Zarzio^®^ | - Binocrit^®^ - Retacrit^®^ | - Omnitrope^®^ | - Abasaglar^®^ | - Bemfola^®^ - Ovaleap^®^ | - Inflectra^®^ - Remsima^®^ - Flixabi^®^ | - Benepali^®^ |
| Germany | - Accofil^®^ - Filgrastim Hexal^®^ - Grastofil^®^ - Nivestim^®^ - Ratiograstim^®^ - Tevagrastim^®^ - Zarzio^®^ | - Abseamed^®^ - Binocrit^®^ - Epoetin Alfa Hexal^®^ - Retacrit^®^ - Silapo^®^ | - Omnitrope^®^ | - Abasaglar^®^ | - Bemfola^®^ - Ovaleap^®^ | - Inflectra^®^ - Remsima^®^ - Flixabi^®^ | - Benepali^®^ |
| Ireland | - Grastofil^®^ - Nivestim^®^ - Ratiograstim^®^ - Tevagrastim^®^ - Zarzio^®^ | - Retacrit^®^ | - Omnitrope^®^ | - Abasaglar^®^ | - Bemfola^®^ | - Inflectra^®^ - Remsima^®^ | - Benepali^®^ |
| Italy | - Nivestim^®^ - Accofil^®^ - Ratiograstim^®^ - Tevagrastim^®^ - Zarzio^®^ | - Binocrit^®^ - Retacrit^®^ | - Omnitrope^®^ | - Abasaglar^®^ | - Bemfola^®^ - Ovaleap^®^ | - Inflectra^®^ - Remsima^®^ | - Benepali^®^ |
| Latvia | - Accofil^®^ - Nivestim^®^ - Tevagrastim^®^ - Zarzio^®^ | - Retacrit^®^ | - Omnitrope^®^ | - | - Ovaleap^®^ | - Inflectra^®^ - Remsima^®^ | - |
| Malta | - Nivestim^®^ | - | - | - | - | - Inflectra^®^ | - |
| Netherlands | - Accofil^®^ - Nivestim^®^ - Tevagrastim^®^ - Zarzio^®^ | - Abseamed^®^ - Binocrit^®^ - Retacrit^®^ | - Omnitrope^®^ | - Abasaglar^®^ | - Bemfola^®^ - Ovaleap^®^ | - Inflectra^®^ - Remsima^®^ - Flixabi^®^ | - Benepali^®^ |
| Poland | - Accofil^®^ - Grastofil^®^ - Nivestim^®^ - Tevagrastim^®^ - Zarzio^®^ | - Binocrit^®^ | - Omnitrope^®^ | - Abasaglar^®^ | - Bemfola^®^ - Ovaleap^®^ | - Inflectra^®^ - Remsima^®^ | - Benepali^®^ |
| Portugal | - Accofil^®^ - Nivestim^®^ - Zarzio^®^ | - Binocrit^®^ - Retacrit^®^ | - Omnitrope^®^ | - Abasaglar^®^ | - Bemfola^®^ - Ovaleap^®^ | - Inflectra^®^ - Remsima^®^ | - Benepali^®^ |
| Slovenia | - Accofil^®^ - Grastofil^®^ - Tevagrastim^®^ - Zarzio^®^ | - Binocrit^®^ | - Omnitrope^®^ | - Abasaglar^®^ | - Bemfola^®^ - Ovaleap^®^ | - Inflectra^®^ - Remsima^®^ | - |
| Spain | - Accofil^®^ - Nivestim^®^ - Ratiograstim^®^ - Tevagrastim^®^ - Zarzio^®^ | - Binocrit^®^ - Epoetin alfa Hexal^®^ - Retacrit^®^ | - Omnitrope^®^ | - Abasaglar^®^ | - Bemfola^®^ - Ovaleap^®^ | - Inflectra^®^ - Remsima^®^ - Flixabi^®^ | - Benepali^®^ |
| Sweden | - Accofil^®^ - Nivestim^®^ - Ratiograstim^®^ - Zarzio^®^ | - (Binocrit^®^: not on the market at the moment, 04/2017) - Retacrit^®^ | - Omnitrope^®^ | - Abasaglar^®^ | - Bemfola^®^ | - Inflectra^®^ - Remsima^®^ | - Benepali^®^ |
| Non-EU countries | | | | | | | |
| Iceland | - Zarzio^®^ | - | - Omnitrope^®^ | - | - Bemfola^®^ | - Inflectra^®^ - Remsima^®^ | - Benepali^®^ |
| Norway | - Nivestim^®^ - Tevagrastim^®^ - Zarzio^®^ | - Retacrit^®^ | - Omnitrope^®^ | - Abasaglar^®^ | - Bemfola^®^ - Ovaleap^®^ | - Inflectra^®^ - Remsima^®^ | - Benepali^®^ |
| Russia | - Tevagrastim^®^ - Zarzio^®^ | - | - Omnitrope^®^ | - | - | - | - |
| Serbia | - Nivestim^®^ - Tevagrastim^®^ - Zarzio^®^ | - Binocrit^®^ | - Omnitrope^®^ | - | - | - Inflectra^®^ - Remsima^®^ | - |
